# Prevalence of non-communicable disease among displaced Rohingya in southern Bangladesh: a first look at a persecuted ethnic minority from Myanmar

**DOI:** 10.1093/inthealth/ihad106

**Published:** 2023-11-01

**Authors:** Jason T Tsichlis, Ipsita Hamid Trisha, Ghazal Aghagoli, Meerjady Sabrina Flora, M Ruhul Abid

**Affiliations:** T he W arren Alpert Medical School of Brown University, 1 Hoppin st, Providence, RI 02903, USA; Health and Education for All, H 31, R 16, Sector 13, Uttrara, Dhaka 1230, Bangladesh; T he W arren Alpert Medical School of Brown University, 1 Hoppin st, Providence, RI 02903, USA; Directorate General of Health Services, Ministry of Health and Family Welfare, Mohakhali, Dhaka 1200, Bangladesh; T he W arren Alpert Medical School of Brown University, 1 Hoppin st, Providence, RI 02903, USA

**Keywords:** Bangladesh, forcibly displaced Myanmar National, global health, non-communicable disease, refugee, Rohingya

## Abstract

**Background:**

In Cox's Bazar, Bangladesh, 860 356 Rohingya living in refugee camps have experienced decades of persecution. Little is known about disease burden in this population.

**Methods:**

A retrospective review of deidentified electronic health records (EHR) of 51 270 Rohingya attending two primary health clinics in Kutupalong and Balukahli from October 2017 to October 2019 was performed. A novel EHR system named NIROG was used for patients' medical records'.

**Results:**

Females comprised 53.8% of patients. The median age of females was 25 y and for males it was 19 y. Prevalence of adult hypertension and diabetes was 14.1% and 11.0%, respectively. Also, 16.6% of children aged <5 y had moderate or severe acute malnutrition, while 36.6% were at risk of malnutrition. Body mass index (BMI) analysis showed that 34.4% of adults were underweight. Females were more likely to be hypertensive, diabetic, overweight/obese and malnourished. BMI had a statistically significant positive correlation with fasting blood glucose levels and systolic blood pressure.

**Conclusions:**

The use of a portable EHR system was highly effective at providing longitudinal care in a humanitarian setting. Significant proportions of the adult population appear to have hypertension or diabetes, pointing to a critical need for management of chronic non-communicable diseases (NCDs). The findings of the current study will help stakeholders to plan effective prevention and management of NCDs among displaced Rohingya and other displaced populations.

## Introduction

The ethnic minority Rohingya have lived in the Rakhine State on the western coast of Myanmar since the eighth century AD.^1^ Numbering approximately 1.5 million, they have been designated as the most persecuted minority in the world by the United Nations (UN),^2,3^ because they have endured prolonged discrimination, marginalization and state-sponsored violence. Since 1948, there have been 13 documented military actions by Myanmar against the Rohingya, including mass executions, destruction of property and places of worship, gender-based violence and forced labor.^4^ Their modern definition of statelessness began in 1982, when Myanmar adopted constitutional language to deny their Burmese citizenship.^5^ As a result of this persistent disenfranchisement, detailed studies of this population's health status have been extraordinarily difficult. In August 2017, state-sponsored violence by the Myanmar army led to the displacement of 745 000 Rohingya into southern Bangladesh.^6^ There are now >1 million displaced Rohingya living in Bangladesh, mostly clustered in the Cox's Bazar and Bandarban regions.^7^ Bangladesh, a lower-middle income country and one of the most densely populated countries in the world, has encountered negative environmental, sociopolitical and financial impacts supporting Rohingya refugees for >5 y.^8,9^

Crowded conditions in the camps, inadequate shelter, emergent communicable disease outbreaks and natural disasters including monsoons and landslides contributed to what the UN described as the fastest growing refugee crisis in the world in 2017.^10^ The displaced Rohingya now live in >34 separate camps that are served by multiple Bangladeshi government ministries, including the Refugee Relief and Repatriation Commissioner (RRRC), the WHO and 146 partners including Bangladeshi non-governmental organizations (NGOs), eight UN agencies and 62 international NGOs.^11^ Undiagnosed and undermanaged non-communicable chronic diseases that require regular monitoring and follow-up treatment, such as hypertension (HTN) and diabetes mellitus (DM), have been identified as growing areas of concern. Medical resources in the camps can be sporadic with limited access to healthcare and a lack of systematic treatment and follow-up.^12^ Psychosocial support has also been identified as a necessary component of health outreach, as displaced Rohingya experience high levels of post-traumatic stress disorder and gender-based violence.^13^

The two camps assessed in this study, known as Kutupalong and Balukhali, are among the largest refugee camps in the world. The persecuted and displaced Rohingya from Myanmar have been continuously settling in these areas for decades.^14^ They have historically been understudied as a population, and a lack of data on their non-communicable diseases (NCDs) and nutritional status greatly hampers public health efforts in the camps. This is the first and most comprehensive retrospective study (N=51 270) of the displaced Rohingya, which analyzed data collected from 100 807 individual patient visits to two health clinics operating in Kutupalong and Balukhali from 9 October 2017 to 3 October 2019. The current study provides details of the NCD burden affecting the Rohingya, while highlighting the mechanisms of a novel healthcare delivery model using a portable electronic health records (EHR) system in a humanitarian crisis.

## Materials and Methods

A retrospective study using deidentified data from the encrypted, Health Insurance Portability and Accountability Act (HIPAA)-compliant, NIROG system of EHR was conducted in collaboration with Health and Education for All (HAEFA), a non-profit organization serving disadvantaged and displaced populations in Bangladesh, Bangladesh Directorate General of Health Services (DGHS) and the Office of the RRRC. The data covered clinical services at the two HAEFA primary health clinics in Kutupalong and Balukhali camps from 9 October 2017 to 3 October 2019, serving displaced Rohingya (N=51 270, a total of 100 826 clinic visits). The study was approved by the Bangladesh DGHS and Brown University's Institutional Review Board, following the World Medical Association Declaration of Helsinki guidelines.

### Study variables

The deidentified data collected for analysis included age, gender, basic anthropometric measurements, blood pressure (BP), heart rate (HR), random blood glucose (RBG), fasting blood glucose (FBG), mid-upper arm circumference (MUAC) when applicable, body mass index (BMI), chief complaints, family history, current medications, passive TB screening and social histories.

### Data collection methods and study criteria

Anthropometric measurements were taken in the standard anatomical position.^15^ BP and HR were measured with digital automated BP machines (ReliOn, Bentonville, AR) while patients were seated. Diagnosis of elevated BP (EBP) and HTN required four BP readings (consecutive systolic and diastolic BPs) from two clinic visits or a previous HTN diagnosis in NIROG. Classifications followed the latest American College of Cardiology and American Heart Association HTN guidelines:

- EBP: systolic BP 120–129 mmHg and diastolic BP≤80 mmHg.
- HTN stage I (HTN1): systolic BP 130–139 mmHg or diastolic BP 80–89 mmHg.
- HTN stage II (HTN2): systolic BP≥140 mmHg or diastolic BP≥90 mmHg.
- Hypertensive urgency: systolic BP>180 mmHg and/or diastolic BP>120 mmHg.^16^

Glucometer (1Touch, Lifescan, Inverness, UK) was used for RBG and FBG measurements obtained through finger pricks. DM diagnosis required FBG≥126 mg/dL after at least 8 h of fasting or an existing DM diagnosis in NIROG. Prediabetes (pre-DM) was determined by FBG 100–125 mg/dL. Hypoglycemia was determined by FBG or RBG ≤70 mg/dL.^17^

MUAC was measured using a standard plastic tape midway between the right olecranon process and acromion. Malnutrition status was determined automatically by NIROG using standard anthropometry criteria.^18^ Weight status relied on WHO z-scores and BMI growth charts in children and adolescents, with categories for underweight, overweight, severely underweight and obese.^19^

TB presumptive cases in NIROG presented a cough lasting >4 wk or two of the following: low-grade evening-rise fever, weight loss and nocturnal diaphoresis.^20^ TB diagnosis was confirmed by acid-fast bacilli (AFB) sputum microscopy at an external government health clinic. Other health information was self-reported and recorded in NIROG. Provisional diagnoses used standard ICD-10 codes in NIROG.

Both adult and pediatric patients received care that was free of charge. The health workers were trained according to a standardized protocol developed by HAEFA to eliminate bias from health delivery and data collection. The EHR system, NIROG,^21^ functioned on a HIPAA-compliant, encrypted, password-protected server that was powered by solar panels. The study used deidentified health data without individually identifiable health information.

### Statistical analysis

Statistical analysis employed Python v. 3.4 and v. 3.6.8 (Pandas and Statsmodels packages) (Python Software Foundation, Wilmington, DE, USA), IBM SPSS v. 26 (IBM, Staten Island, NY, USA) and MedCalc v. 19.5.1 (MedCalc Software Ltd., Ostend, Belgium). Independent t-tests assessed age differences between genders. Wald tests determined OR significance for gender differences in HTN, DM, malnutrition, BMI status and areca nut consumption. Linear regression analyzed the relationship between BMI with FBG and systolic BP, while logistic regression assessed the relationship between systolic BP and DM. χ^2^ analyses compared female and male prevalence for select provisional diagnoses, EBP, HTN, pre-DM, DM, nutritional status, BMI status and areca nut consumption. Due to the large sample size of all compared cohorts, the power of all OR analyses and linear and logistic regressions was found to be 1.00.^22^

## Results

### Demographics

Among 51 270 patients who visited the HAEFA health clinics at Kutupalong and Balukhali, 53.8% (n=27 600) were female and 46.2% (n=23 670) were male (Table 1). The average self-reported age for females was 26.29±18.02 y and 25.73±33.57 y for males. The median age was 25 y for females and 19 y for males.

**Table 1.** Demographics of patients by camp

	N			Mean age, y			Median age, y		
	Total	Female (%)	Male (%)	Total (SD)	Female (SD)	Male (SD)	Total	Female	Male
Kutupalong Camp	24 157	13 570 (56.2)	10 587 (43.8)	26.08 (±20.20)	26.53 (±18.28)	25.50 (±22.40)	24	25	19
Balukhali Camp	27 113	14 030 (51.8)	13 083 (48.3)	25.74 (±19.11)	26.11 (±17.71)	25.36 (±20.50)	23	25	20
Total	51 270	27 600 (53.8)	23 670 (46.2)	26.03 (±26.37)	26.29 (±18.02)	25.73 (±33.57)	23	25	19

The demographics of the Kutupalong and Balukhali camps are similar. In total, females make up 53.8% of the population, whereas males comprise 46.2% of the population. The mean age for females is 26.29 y and 25.73 y for males, with a median age for females of 25 y and 19 y for males.

### Most common provisional diagnoses

Five provisional diagnoses amounted to 50.3% of all diagnosed patients (Table S1): nasopharyngitis (14.6%, n=7488), unspecified fever (13.1%, n=6697), dyspepsia (10.0%, n=5101), arthritis (6.3%, n=3253) and diarrhea (6.3%, n=3247). More males were diagnosed with nasopharyngitis (12.8% female, 16.7% male; [N-1]*X^2^*=148.35, p<0.001), unspecified fever (11.6% female, 14.8% male; [N-1]*X^2^*=100.24, p<0.001) and diarrhea (5.5% female, 7.4% male; [N-1]*X^2^*=76.74, p<0.001). More females were diagnosed with dyspepsia (11.4% female, 8.3% male; [N-1]*X^2^*=137.62, p<0.001) and arthritis (7.0% female, 5.58% male; [N-1]*X^2^*=42.67, p<0.001).

### Prevalence of hypertension

**Figure 1.**
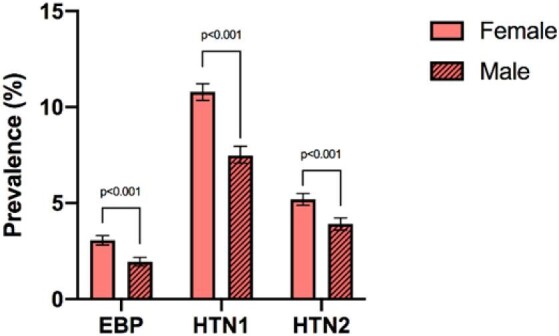
The prevalence of elevated blood pressure (EBP) was 2.6% (3.1% in women and 1.9% in men), for hypertension 1 (HTN1) was 9.4% (10.8% in women and 7.5% in men) and for hypertension 2 (HTN2) was 4.7% (5.2% in women and 3.9% in men). The prevalence of EBP, HTN1 and HTN2 was significantly greater in females than males. p<0.001.

HTN prevalence in patients aged ≥12 y was 14.1% (Figure 1, Table S2). The prevalence of HTN was 16.0% in females and 11.4% in males ([N-1]*X^2^*=142.13, p<0.001). The age group with the highest prevalence of HTN was 65–80 y (30.3%; 29.4% in females and 30.6% in males). The prevalence of EBP among the total population was 2.6% (Table S3). The prevalence of EBP was 3.1% in females and 1.9% in males ([N-1]*X^2^* =105.30, p<0.001). The age group with the most cases of EBP in females was 18–40 y (67.4%) and 40–65 y in males (55.5%).

### Prevalence of DM and impaired blood glucose levels

**Figure 2.**
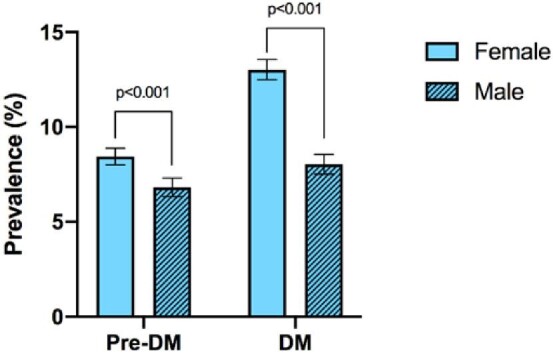
The prevalence of prediabetes mellitus (pre-DM) was 7.8% (8.45% in women and 6.8% in men). The overall prevalence of diabetes mellitus (DM) was 11.0% (13.02% in females and 8.0% in males). The prevalence of both pre-DM and DM was significantly greater in females than males. p<0.001.

DM prevalence was 11.0% (Table S4). The prevalence of DM was 13.0% in females and 8.0% in males ([N-1]*X^2^*=156.16, p<0.001) (Figure 2). The age group with the highest prevalence of DM in females was 40–65 y (19.0%) and in males was 65–80 y (15.5%). The prevalence of pre-DM was 7.8% (Table S5). The prevalence of pre-DM was 8.5% among females and 6.8% among males ([N-1]*X^2^*=22.81, p<0.001). The age group with the most cases of pre-DM in females was 18–40 y (54.2%) and 40–65 y in males (49.2%). Hypoglycemia was noted in 2.8% of patients and there was no statistically significant difference between genders. The age group with the most cases of hypoglycemia in both females and males was 18–40 y (78.1% and 55.4%, respectively) (Table S5).

### Nutrition status in children aged <5 y

**Figure 3.**
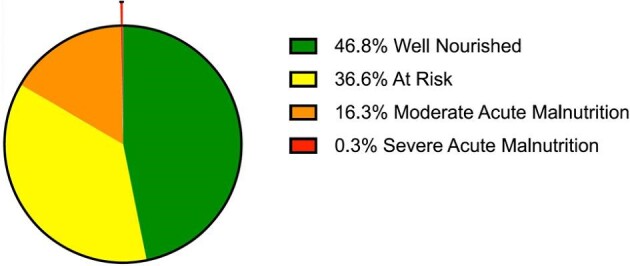
In total, 16.3% of children had moderate acute malnutrition (orange), 0.3% had severe acute malnutrition (red) and 36.6% of children were at risk of malnutrition (yellow); 46.8% of children aged <5 y were well nourished (green); N=4544.

Of 4544 children measured, 46.8% of children aged <5 y were well nourished, 36.6% were at risk of malnutrition, 16.3% had moderate acute malnutrition (MAM) and 0.3% had severe acute malnutrition (SAM) (Figure 3 and Table S6). Overall, 57.1% of all females were at risk of malnutrition or were diagnosed with MAM or SAM compared with 49.9% of males ([N-1]*X^2^*=23.34, p<0.001).

### BMI

The mean BMI was 19.25±4.75 kg/m^2^ (legend of Table S7 and Figure S1). From the analyzed population, 53.4% had normal weight, 21.3% were underweight, 13.1% were severely underweight, 9.6% were overweight and 2.7% were obese (Figure S1). In both females and males, a majority of patients (56.5% of females and 49.6% of males) had normal weight (Table S7). Also, 16.6% of females and 26.9% of males were underweight. Females had a higher prevalence of overweight or obese status (16.5% and 7.2% prevalence, respectively; [N-1]*X^2^*=939.37, p<0.001), while males had a higher prevalence of underweight or severely underweight status (43.2% and 27.0% prevalence, respectively; [N-1]*X^2^*=1341.37, p<0.001) (Table S7).

### TB

There were 657 presumptive TB cases (48.9% female and 51.1% male). Also, 158 patients provided sputum for AFB microscopy. Three cases were diagnosed to have TB (1.9%) and were treated with anti-TB drugs (Table S9).

### Areca (betel) nut consumption

In total, 9043 individuals aged >15 y reported consumption of areca nuts (prevalence=28.3%; [N-1] *X^2^*=23.65, p<0.001) (Table S10). Areca nut consumption was associated with 1.55 times higher odds of DM (95% CI 1.43 to 1.70, p<0.001) (Table S11).

### Females were more likely than males to have a diagnosis of HTN and DM

Females were more likely than males to have a diagnosis of HTN (OR 1.48; 95% CI 1.39 to 1.58) and DM (OR 1.71; 95% CI 1.57 to 1.87) (Table 2). Females aged <5 y were at a greater risk of MAM and SAM (OR 1.60; 95% CI 1.36 to 1.87). Obesity was more likely to be present in females (OR 2.56; 95% CI 2.41 to 2.72, respectively), while males were more likely to be underweight/severely underweight (OR 0.49; 95% CI 0.47 to 0.51).

**Table 2.** Gender differences in HTN, DM, nutrition status and BMI

				95% CI		
	Female prevalence, %	Male prevalence, %	OR	−	+	p
HTN1+2^a^	16	11.4	1.48	1.39	1.58	<0.001
DM^b^	13.0	8.0	1.71	1.57	1.87	<0.001
MAM+SAM^c^	20.0	13.6	1.60	1.36	1.87	<0.001
Underweight and severely underweight^d^	27.0	43.2	0.49	0.47	0.51	<0.001
Overweight and obese^d^	16.5	7.2	2.56	2.41	2.72	<0.001

Females had higher odds of being hypertensive (OR 1.48, 95% CI 1.39 to 1.58) and diabetic (OR 1.71, 95% CI 1.57 to 1.87) than males. Females aged 6–59 mo were more likely to have either moderate or severe acute malnutrition than males (OR 1.60, 95% CI 1.36 to 1.87). Females aged >2 y were less likely to be underweight or severely underweight (OR 0.49, 95% CI 0.47 to 0.51) and were more likely to be overweight or obese (OR 2.56, 95% CI 2.41 to 2.72).

a Hypertension type 1 (HTN1) and hypertension type 2 (HTN2).

b Diabetes mellitus (DM).

c Moderate acute malnutrition (MAM) and severe acute malnutrition (SAM) as measured by mid-upper arm circumference (MUAC) in children aged 6–59 mo.

d In patients aged ≥2 y. Determined by body mass index (BMI) measurement.

### Regression analyses

BMI had a statistically significant positive correlation with FBG (r=0.11, p<0.001) and systolic BP (r=0.18, p<0.001), suggesting a direct association of increased BMI with higher FBG and systolic BP (Table S12). DM had a statistically significant yet clinically insignificant negative correlation with systolic BP (r=−0.02, p<0.001). There were no appreciable gender differences in the above regressions.

## Discussion

This retrospective review of deidentified EHR is among the first to comprehensively assess the health status of 51 270 Rohingya living in two of the largest refugee camps, Kutupalong and Balukhali, in Cox's Bazar, Bangladesh. This disease prevalence analysis has a particular emphasis on NCDs, which are difficult to follow and are often neglected in humanitarian crises due to a lack of appropriate individualized EHR and follow-up systems.

The patient population at the HAEFA primary health clinics in Kutupalong and Balukhali refugee camps is predominantly young, with a median age of 25 y for females and of 19 y for males. Nearly 72% of patients in these camps are aged <40 y, reflecting a young population with potential to grow. Protracted persecution of the Rohingya in Myanmar also contributes to the population in the camps skewing young.^23^ The clinics have been successful in engaging female patients, comprising more than one-half of the clinic's visitors. The camps, themselves, however, are already at environmental capacity, and due to the deforestation required to create the space necessary, the area is acutely vulnerable to flooding, fire and other natural disasters.^24^ Additionally, close-quarter living conditions can exacerbate disease spread and overall health challenges.

Regarding health conditions, the prevalence of hypertension (HTN1 and HTN2 combined) among Rohingya refugees is lower than among Bangladeshi adults (14.1% vs 40.7%).^25^ This lower prevalence can be attributed to factors such as a younger screening age in our study (≥12 y), a younger median age, lifestyle and dietary variation, as well as potential ethnic differences. Conversely, HTN2 prevalence in Rohingya refugees is higher than HTN2 among Burmese adults aged >15 y (4.7% vs 3.8%).^26^ This higher prevalence may be linked to factors resulting from prolonged persecution, such as mental health challenges and post-traumatic stress disorder.^26^ Additionally, as the Rohingya are a unique and historically isolated population, the discrepancies between their HTN prevalence and that of the Bangladeshis and Burmese could possibly also be attributed to founder effect, or a genetic bottleneck given repeated mass casualty events in their recent history. Similar effects are seen in other distinct communities living within larger, less homogeneous populations, as exemplified by the American Amish.^25^ Research on genetic underpinnings of hypertension and population-level allele variance in these populations is needed.

Early detection of diabetes can lead to better patient outcomes by better blood glucose management through lifestyle changes or medical treatment.^27^ Our data suggest that, compared with the overall Bangladeshi and Myanmar population, the Rohingya refugees have a higher prevalence of DM and a lower prevalence of pre-DM.^28,29^ The same factors of ethnicity, genetics, history of persecution, camp conditions and lifestyle may have contributed to the higher prevalence of DM in the Rohingya population. While diet differences such as grains and processed foods within the camps could have contributed to the differences in prevalence, our study could not conduct HbA1c studies due to cost constraints. Managing NCDs is difficult in humanitarian crises due to logistical challenges, limited resources for care of chronic cases and barriers to healthcare,^30^ leading to a higher prevalence of unmanaged DM.

BMI is an important factor known to contribute to the higher prevalence of HTN and DM.^31^ Of the 46 526 individuals analyzed, 53.4% had normal BMI, 21.3% were underweight, 13.1% were severely underweight, 9.6% were overweight and 2.7% were obese. Our data revealed a statistically significant positive correlation between BMI and FBG as well as systolic BP among the Rohingya. The rate of overweight individuals is higher in Bangladesh and Myanmar compared with Rohingya refugees. The WHO reports the prevalence of being overweight and obese as 17.0% and 3.3%, respectively, in Bangladesh, and as 17.4% and 2.9%, respectively, in Myanmar.^29^ Limited resources within the camps could play a role in this discrepancy.

Of note, a correlation between areca nut consumption and DM among the Rohingya was identified: 28.3% of Rohingya refugees aged >15 y reported consuming areca nuts. Our data demonstrated that areca nut consumption was associated with a statistically significant risk of DM with an OR of 1.55 (95% CI 1.43 to 1.70, p<0.001). These findings support prior research linking areca nut consumption with increased risks of DM and HTN.^32^ As there are sparse NCD data on this population, these findings help to illustrate potential contributing factors to their NCD burden.

In addition to EBP and hyperglycemia, three of the five most common provisional diagnoses were related to infectious disease processes. Nasopharyngitis and diarrhea were common with an incidence of 14.61% and 6.3%, respectively. In Bangladesh, the top two infectious diseases (according to a WHO report in 2019) were diarrhea (16%) and respiratory infections (7.7%).^10^ The tenuous nature of housing, overcrowding and common latrines may serve as predominant factors contributing to infectious diseases in the camps. Monsoon seasons often lead to floods and prevent access to safe drinking water.^24,33^

HAEFA provides passive TB screening for all patients at each visit. In 2018, an estimated 364 000 TB cases were reported in Bangladesh, with a prevalence of about 0.2%. Myanmar had an even higher prevalence, with 191 000 cases reported in 2018 (prevalence=0.4%).^34,35^ Our findings show a higher prevalence of TB among displaced Rohingya (1.9%), which requires further investigation. As a slow-growing pathogen, TB is transmitted most effectively in crowded conditions among immunocompromised individuals. Although the WHO reports a 95% success rate in TB treatment among the Rohingya in Bangladesh, COVID-19 has negatively affected screening and directly observed treatment within the camps,^36^ raising the specter of drug resistance.

Children were assessed for their nutrition status (n=4544), and the majority were not adequately nourished (53.2%). Specifically, 36.6% were at risk of malnutrition, 16.3% had MAM and 0.3% had SAM. The prevalence of malnutrition was higher in females than in males across all categories. Malnutrition in children has many adverse outcomes, including irreversible damage to biological and cognitive development.^37^ Malnutrition also increases the risk of DM, obesity and cardiovascular diseases.^38^ Interestingly, adult females in the camps were found to be more likely to be overweight, which could be secondary to malnutrition in childhood.

This study is not without limitations. HAEFA clinics provide primary care and are limited by resources, such as specialty care and tests, including oral glucose tolerance test or HbA1c, limiting insight into DM. Similarly, due to limitations on hemoglobin measurement, our examination of anemia was also limited. Additionally, some data were missing, possibly due to issues with local internet/Wi-Fi or solar power of the EHR system, which was accounted for in data analysis. Lastly, the dataset in its current format did not allow for assessment of long-term treatments and outcomes of chronic conditions. There are future plans to address this limitation by evaluating NCD treatment efficacy in this population.

### Conclusions

This study comprehensively assessed the health status of displaced Rohingya living in Bangladesh and, to date, is the largest systematic study of this population. Significant proportions of the adult population appear to have hypertension or diabetes, pointing to a critical need for management of chronic non-communicable diseases (NCD). The establishment of HAEFA clinics, in coordination with the DGHS and RRRC, and an on-site EHR system (i.e. NIROG), have enabled follow-up and longitudinal tracking of patients’ records for NCD management. Studies like this one are important for identifying the health status of large refugee populations and addressing their healthcare needs with a special focus on NCDs. There are currently >89 million displaced people in the world.^39,40^ As the average length of refugee displacement in the world is now >20 y, the UN and international organizations should support local government and healthcare organizations to provide effective screening and management for NCDs. This may help reduce mortality and long-term disability given the current trend of protracted refugee situations worldwide.

## Supplementary Material

ihad106_Supplemental_File

## Data Availability

The processed, deidentified data collected during the study period will be made available alongside a data dictionary with publication. Additionally, analytic code will be made available at the time of publication. Due to the nature of the study (retrospective review) and the protected nature of the participants, all deidentified raw and processed data and analytic code that were used in this study will be available for researchers who provide ethical proposals with valid methodology. Proposals should be addressed to ruhul_abid@brown.edu and a data access agreement will be signed by approved parties. This data will subsequently be made available for the amount of time necessary for the purposes of the agreed upon study.
