# Supplementary material for: Prevalence of non-communicable disease among displaced Rohingya in southern Bangladesh: a first look at a persecuted ethnic minority from Myanmar
Source: Int Health. 2023 Nov 1;16(4):409–15. doi: 10.1093/inthealth/ihad106 (PMC11218878; doi:10.1093/inthealth/ihad106)
Supplement: ihad106_Supplemental_File [file ihad106_supplemental_file.docx]

**Supplementary Appendix**

**Prevalence of Non-Communicable Disease among Displaced Rohingya in Southern Bangladesh: A First Look at a Persecuted Ethnic Minority from Myanmar**

Jason T. Tsichlis, MD, MS^a,1^; Ipsita Hamid Trisha, MBBS, MPH^b,2^; Ghazal Aghagoli, ScB^a^; Meerjady Sabrina Flora, MBBS, MPH, PhD^c^; M. Ruhul Abid, MD, PhD^a*^

^a^The Warren Alpert Medical School of Brown University, Providence, RI, USA;

^b^Health and Education for All, Dhaka, Bangladesh;

^c^Directorate General of Health Services, Ministry of Health and Family Welfare, Dhaka,

Bangladesh

^1^Current address: Department of Pediatrics, University of California, San Francisco School of Medicine, San Francisco, CA, USA

^2^Current address: Department of Neurology, University of Arizona, University of Arizona College of Medicine, Tucson, AZ, USA

*Corresponding author: Tel: 401-444-6527; E-mail: Ruhul_Abid@brown.edu

**Table of Contents**

1. Author Reflexivity Statement……………………………………………………………………………3
2. Supplementary Method*s*…………………………………………………………………………………5
   1. Patient and public involvement……………………………………………………………………..5
   2. Management of missing data and analysis thresholds……………………………………………...5
   3. Study variables not included in data analysis………………………………………………………5
3. Supplementary Tables and Figures……………………………………………………………………...7
   1. Table S1. Five most common provisional diagnoses by sex……………………………………….6
   2. Table S2. Age-stratified HTN1+2^a^ prevalence by sex……………………………………………...6
   3. Table S3. EBP and HTN by age and sex……………………………………………………………7
   4. Table S4. Age-stratified DM prevalence by sex……………………………………………………7
   5. Table S5. DM and Pre-DM by age and sex…………………………………………………………8
   6. Table S6. Nutrition status of children under five years old by MUAC………………………….....9
   7. Table S7. BMI analysis of patients over two years old…………………………………………….9
   8. Figure S1. BMI Analysis……………………………………………………………………………9
   9. Table S8. Clinical anemia prevalence stratified by age and sex…………………………………..10
   10. Table S9. Presumptive TB cases, sputum samples, and confirmed diagnoses……………………10
   11. Table S10. Areca nut consumption, self-reported in patients over 15 years old…………………..10
   12. Table S11. DM risk with areca nut exposure……………………………………………………...11
   13. Table S12. Regression analyses…………………………………………………………………...11
4. References……………………………………………………………………………………………...12

**I. Author Reflexivity Statement**

**1. How does this study address local research and policy priorities**

The study was designed to investigate the non-communicable disease (NCD) burden of the displaced Rohingya population living in two refugee camps in southern Bangladesh. It is the first and largest study of its kind in this population. In addition to assessing the disease burden of common non-communicable diseases, the study acted as a proof-of-concept model for a novel mobile electronic health record (EHR) system used in humanitarian situations. The information and lessons learned from the study will aid local policy makers at the Directorate General of Health Services (DGHS) of Bangladesh Ministry of Health and international and local non-governmental organizations (NGO) in resource allocation and programmatic foci when administering services to this highly vulnerable population.

**2. How were local researchers involved in study design?**

The study design was conceived in conjunction with the corresponding author (MRA), fourth author (MSF), and the second author (IHT), who are from the country of study. The mobile EHR that was used for data collection and output of de-identified patient data was developed by a Bangladeshi software development company. All data was collected by a team of Bangladeshi doctors, nurses, and healthcare workers who were paid for their work.

**3. How has funding been used to support the local research team?**

This study was an unfunded retrospective analysis of de-identified patient data. The organization that compiled the data, Health and Education for All (HAEFA), is a leading local NGO working in two of the largest refugee camps. They employ all Bangladeshi nationals for clinical, administrative, and logistical efforts. All employees are fully compensated by HAEFA for their labor with competitive pay.

**4. How are research staff who conducted data collection acknowledged?**

The healthcare workers who collected the data as part of their clinical duties in patient management are acknowledged in the Acknowledgements section of the manuscript.

**5. Do all members of the research partnership have access to the study data?**

All members of the partnership have access to the data.

**6. How was data used to develop analytical skills within the partnership?**

JTT and IHT guided the data analysis of the raw data with help from partners acknowledged on the paper. All authors were involved in the analytical interpretation.

**7. How have research partners collaborated in interpreting study data?**

Regular communication was maintained between all authors on the study through the data analysis and interpretation process.

**8. How were research partners supported to develop writing skills?**

As the senior author on the manuscript, MRA supported JTT, IHT, and GA in the writing process.

**9. How will research products be shared to address local needs?**

As mentioned briefly in response to question 1, the products and conclusions drawn from this paper will be useful for resource allocation pertaining to the massive response required to address the health and wellbeing of the displaced Rohingya community in southern Bangladesh. The implications of the mobile EHR, however, extend beyond the Rohingya population and show promise for being applied in other low-resource setting or humanitarian crisis.

**10. How is the leadership, contribution, and ownership of this work by LMIC researchers recognized within the authorship?**

The majority of the authors (IHT, MSF, and MRA) are from Bangladesh, the LMIC country of focus for the study.

**11. How have early career researchers across the partnership been included within the authorship team?**

The first author, JTT, was a medical student at time of data analysis and most of the manuscript writing. IHT and JTT are now medical residents. GA is currently a medical student.

**12. How has gender balance been addressed within the authorship?**

The majority of the authors are female (IHT, GA, and MSF).

**13. How has the project contributed to training of LMIC researchers?**

The authorship team is primarily composed of researchers from Bangladesh. Two senior researchers (MSF and MRA) are from Bangladesh. The second author, IHT, is also from Bangladesh and has been trained by MSF and MRA.

**14. How has the project contributed to improvements in local infrastructure?**

The project is a retrospective data analysis and does not have any direct impacts on local infrastructure. However, conclusions from the study have several recommendations for infrastructural improvement as pertaining to the medical infrastructure in the refugee camps assessed. Upon publication, this study may play a major role in the improvement of local health infrastructure in the refugee camps to manage NCD and their complications. Other organizations and NGO working in the camps will also be benefitted from the findings and recommendations of this study.

**15. What safeguarding procedures were used to protect local study participants and researchers?**

All data was collected in private consultation with medical professionals as part of their clinical service at health clinics within the largest refugee camps in southern Bangladesh. The data was stored in a HIPAA-compliant, encrypted, password-protected Microsoft azure server. All data was extracted in de-identified format without any PHI and stored in a password-protected folder on a password-protected computer. Please see methods for more specifics.

**II. Supplementary Methods**

**a. Patient and Public Involvement**

Per the nature of this retrospective study using de-identified patient data, the study participants were not involved in the planning, research gathering, or analysis—and the study did not impose any burdens on the participants themselves, as their data was collected during regular clinic visits. However, their participation in seeking medical care at HAEFA clinics involved informed consent about establishing an EHR account within the NIROG system. Patients were made aware that their de-identified data could be used for potential research. HAEFA incorporates Rohingya community liaisons that spread information about the clinics; these liaisons were not used in recruitment specifically for the study.

**b. Management of missing data and analysis thresholds**

From the initial dataset of 51,289 patients seen in 100,826 individual visits, 34 unique visits were without age data. Of those 34 visits, 15 patients returned to the clinic at least one more time and provided age data in subsequent visits and all of their visits were included in the analysis. The remaining 19 patients (five from Balukhali and 14 from Kutupalong) did not return to the clinic and thus were eliminated from analysis, leaving a total of 51,270 patients in 100,807 individual visits. In all analyses, missing data were deemed missing completely at random, as no relationship between the missingness of these data and any values could be identified. All missing values for variables other than age were treated similarly and were subsequently eliminated from their respective analyses.

With the size of the study population, and lack of characterization of this population in epidemiological literature, small amounts of biologically implausible values were cleaned from continuous scale data and excluded from analyses using methods validated in other studies as described below [1]. BP was measured in a total of 33,652 patients above 12-years-old. For all diastolic BP readings <40mmHg and greater than >130mmHg were discarded. All systolic BP readings  <65mmHg and  >230mmHg were discarded. In total, 31 patients were excluded, leaving 33,621 patients’ data [2]. Blood glucose measurements were assessed in 25,634 patients. Definitive diagnosis of DM was made based on FBG, and RBG in cases where FBG values were not available, and provisional diagnosis in NIROG (ICD-10 code: E11). For both female and male patients of all ages, both FBG and RBG values < 50 mg/dL and  > 600 mg/dL were discarded [3], excluding a total of 44 patients’ records from analysis, leaving 25,590 patients. MUAC was calculated using standard MUAC tape for 59.6% of children aged six to 59 months (4,553 out of 7,643). A total of nine MUAC values below 1.00 cm and above 26.50 cm were discarded [4], leaving 4,544 for definitive analysis. BMI was calculated automatically by the NIROG EMR using measured weight and height in 47,948 patients. Applying the three-sigma rule, BMI values outside three standard deviations from the mean (19.25 ± 4.75 kg/m^3^) for both female and male patients of all ages were discarded: 888 patients with BMI < 4.99 mg/kg^3^ (including 838 values of 0) and 534 patients with BMI  >33.51 mg/kg^3^were eliminated from analysis (Table S14) [4]. In total, 46,526 patients were included in BMI analysis.

**c. Study variables not included in data analysis**

Due to cost and feasibility constraints, anemia was assessed by lower palpebral eyelid test and graded as mild (1+), moderate (2+) or severe (3+) [5] for all patients visiting the clinics, and by hemoglobinometer (HemoCue, USA) only when deemed necessary (e.g. pregnant women, malnourished patients) by clinic medical staff (Table S8). Therefore, while clinically useful, anemia status was not included in this analysis in order to exclude any user bias with clinical diagnosis.

**III. Supplementary Tables and Figures**

**Table S1. Five most common provisional diagnoses by sex.**

|  | **Female**  **(n = 27600)** | | **Male**  **(n = 23670)** | | **Total**  **(N = 51270)** | |
| --- | --- | --- | --- | --- | --- | --- |
|  | **Cases** | **Incidence, %** | **Cases** | **Incidence, %** | **Cases** | **Incidence, %** |
| **Nasopharyngitis** | 3544 | 12.8 | 3944 | 16.7 | 7488 | 14.6 |
| **Unspecified Fever** | 3207 | 11.6 | 3490 | 14.8 | 6697 | 13.1 |
| **Dyspepsia** | 3143 | 11.4 | 1958 | 8.3 | 5101 | 10.0 |
| **Arthritis** | 1932 | 7.0 | 1321 | 5.6 | 3253 | 6.3 |
| **Diarrhea** | 1507 | 5.5 | 1740 | 7.4 | 3247 | 6.3 |

The most common provisional diagnoses included nasopharyngitis, unspecified fever, dyspepsia, arthritis, and diarrhea (total incidences: 14.6%, 13.1%, 10.0%, 6.3%, and 6.3%, respectively). Females had a higher incidence of dyspepsia ([N-1]*X^2^* =137.62, p < 0.001) and arthritis ([N-1]*X^2^* =42.67, p < 0.001), while males had a higher incidence of nasopharyngitis ([N-1]*X^2^* =148.35, p < 0.001), unspecified fever ([N-1]*X^2^* =109.24, p < 0.001), and diarrhea ([N-1]*X^2^* =76.74, p < 0.001).

**Table S2. HTN1+2 prevalence stratified by sex.**

|  | **Female** | | | **Male** | | | **Total**  **(N = 33621)** |
| --- | --- | --- | --- | --- | --- | --- | --- |
| **Age, y** | **Positive Cases** | **Number Screened** | **Prevalence, %** | **Positive Cases** | **Number Screened** | **Prevalence, %** | **Prevalence, %** |
| **12 - <18** | 75 | 1495 | 5.0 | 20 | 1667 | 1.2 | 3.0 |
| **18 - 40** | 1588 | 12101 | 13.1 | 364 | 6410 | 5.7 | 10.6 |
| **>40 - 65** | 1357 | 5565 | 24.4 | 943 | 5108 | 18.5 | 21.6 |
| **>65 - 80** | 98 | 333 | 29.4 | 259 | 846 | 30.6 | 30.3 |
| **>80** | 2 | 26 | 7.7 | 21 | 70 | 30.0 | 24.0 |
| **Total** | 3120 | 19520 | 16.0 | 1607 | 14101 | 11.4 | 14.1 |

The overall prevalence of combined HTN1 and HTN2 was 14.1% (16.0% in females and 11.4% in males). In age groups 12-<18 years, 18-40 years, and >40-65 years, females had higher rates of combined HTN1 and HTN2 than males (5.0% vs 1.2%, 13.1% vs 5.7%, 24.4% vs 18.5%, respectively). Males >65-80 years and >80-years had higher rates than females (30.6% vs 29.4% and 30.0% vs 7.7%, respectively).

**Table S3. EBP, HTN1, and HTN2 prevalence stratified by age.**

|  | **Female (n = 19520)** | | | | | | **Male (n = 14101)** | | | | | |
| --- | --- | --- | --- | --- | --- | --- | --- | --- | --- | --- | --- | --- |
|  | **EBP** | | **HTN1** | | **HTN2** | | **EBP** | | **HTN1** | | **HTN2** | |
| **Age, y** | **Cases** | **% of Total** | **Cases** | **% of Total** | **Cases** | **% of Total** | **Cases** | **% of Total** | **Cases** | **% of Total** | **Cases** | **% of Total** |
| **12 – <18**  **(n_f_ = 1495)**  **(n_m_ = 1667)** | 28 | 4.7 | 70 | 3.3 | 5 | 0.5 | 4 | 1.5 | 19 | 1.8 | 1 | 0.2 |
| **18 – 40**  **(n_f_ = 12101)**  **(n_m_ = 6410)** | 403 | 67.4 | 1177 | 55.9 | 411 | 40.5 | 95 | 34.7 | 299 | 28.3 | 65 | 11.8 |
| **>40 – 65**  **(n_f_ = 5565)**  **(n_m_ = 5108)** | 162 | 27.1 | 806 | 38.3 | 551 | 54.3 | 152 | 55.5 | 581 | 55.1 | 362 | 65.6 |
| **>65 – 80**  **(n_f_ = 333)**  **(n_m_ = 846)** | 5 | 0.8 | 52 | 2.5 | 46 | 4.5 | 22 | 8.0 | 142 | 13.5 | 117 | 21.2 |
| **>80**  **(n_f_ = 26)**  **(n_m_ = 70)** | 0 | 0.0 | 1 | 0.1 | 1 | 0.1 | 1 | 0.4 | 14 | 1.3 | 7 | 1.3 |
| **Total** | 598 | 100.0 | 2106 | 100.0 | 1014 | 100.0 | 274 | 100.0 | 1055 | 100.0 | 552 | 100.0 |

The prevalence of EBP was 2.6% (3.1% in females and 1.9% in males). Of the female cases of EBP, females 18-40-years-old had the most cases (403, 67.4% of female total). Of the male cases of EBP, males >40-65-years-old had the most cases (152, 55.5% of male total). The prevalence of HTN1 was 9.4% (10.8% in females and 7.5% in males). Of the female cases of HTN1, females 18-40-years old had the most cases (1,177, 55.9% of female total). Of the male cases of EBP, males >40-65-years-old had the most cases (581, 55.1% of male total). The prevalence of HTN2 was 4.7% (5.2% in females and 3.9% in males). Of the female cases of HTN2, females >40-56-years-old had the most cases (551, 54.3% of female total). Of the male cases of HTN2, males >40-65-years-old had the most cases (362, 65.6%).

**Table S4. DM prevalence stratified by sex.**

|  | **Female** | | | **Male** | | | **Total**  **(N = 25590)** |
| --- | --- | --- | --- | --- | --- | --- | --- |
| **Age, y** | **Positive Cases** | **Number Screened** | **Prevalence, %** | **Positive Cases** | **Number Screened** | **Prevalence, %** | **Prevalence, %** |
| **10 - <18** | 27 | 1170 | 2.3 | 24 | 1396 | 1.7 | 2.0 |
| **18 - 40** | 1062 | 9318 | 11.4 | 245 | 4530 | 5.4 | 9.4 |
| **>40 - 65** | 848 | 4462 | 19.0 | 460 | 3734 | 12.3 | 16.0 |
| **>65 - 80** | 46 | 260 | 17.7 | 101 | 650 | 15.5 | 16.2 |
| **>80** | 0 | 17 | 0.0 | 3 | 53 | 5.7 | 4.3 |
| **Total** | 1983 | 15227 | 13.0 | 833 | 10363 | 8.0 | 11.0 |

The overall prevalence of DM was 11.0% (13.0% in females and 8.0% in males). In age groups 10-<18 years, 18-40 years, >40-65 years, and >65-80 years, females had higher rates of DM than males (2.3% vs 1.7%, 11.4% vs 5.4%, 19.0% vs 12.3%, and 17.7% vs 15.5%, respectively). Males >80-years-old had DM rate of 5.7% while there were no cases of DM in females over 80-years-old.

**Table S5. Hypoglycemia, pre-DM, and DM prevalence stratified by age**

|  | **Female (n = 15227)** | | | | | | **Male (n = 10363)** | | | | | |
| --- | --- | --- | --- | --- | --- | --- | --- | --- | --- | --- | --- | --- |
|  | **Hypoglycemic** | | **Pre-DM** | | **DM** | | **Hypoglycemic** | | **Pre-DM** | | **DM** | |
| **Age, y** | **Cases** | **% of Total** | **Cases** | **% of Total** | **Cases** | **% of Total** | **Cases** | **% of Total** | **Cases** | **% of Total** | **Cases** | **% of Total** |
| **10 – <18**  **(n_f_ = 1170)**  **(n_m_ = 1396)** | 36 | 8.0 | 58 | 4.5 | 27 | 1.4 | 23 | 8.3 | 45 | 6.4 | 24 | 2.9 |
| **18 – 40**  **(n_f_ = 9318)**  **(n_m_ = 4530)** | 352 | 78.1 | 697 | 54.2 | 1062 | 53.6 | 154 | 55.4 | 249 | 35.2 | 245 | 29.4 |
| **>40 – 65**  **(n_f_ = 4462)**  **(n_m_ = 3734)** | 61 | 13.5 | 493 | 38.3 | 848 | 42.8 | 86 | 30.9 | 348 | 49.2 | 460 | 55.2 |
| **>65 – 80**  **(n_f_ = 260)**  **(n_m_ = 650)** | 2 | 0.4 | 35 | 2.7 | 46 | 2.3 | 15 | 5.4 | 58 | 8.2 | 101 | 12.1 |
| **>80**  **(n_f_ = 17)**  **(n_m_ = 53)** | 0 | 0.0 | 3 | 0.2 | 0 | 0.0 | 0 | 0.0 | 7 | 1.0 | 3 | 0.4 |
| **Total** | 451 | 100.0 | 1286 | 100.0 | 1983 | 100.0 | 278 | 100.0 | 707 | 100.0 | 833 | 100.0 |

The prevalence of hypoglycemia was 2.9% (3.0% in females and 2.7% in males). Of the female cases of hypoglycemia, females 18-40-years-old had the most cases (352, 78.1% of female total). Of the male cases of hypoglycemia, males 18-40-years-old had the most cases (154, 55.4% of male total). The prevalence of pre-DM was 7.8% (8.5% in feamales and 6.8% in males). Of the female cases of pre-DM, females 18-40-years old had the most cases (697, 54.2% of female total). Of the male cases of pre-DM, males >40-65-years-old had the most cases (348, 49.2% of male total). The prevalence of DM was 11.0% (13.0% in females and 8.0% in males). Of the female cases of DM, females 18-40-years-old had the most cases (1,062, 53.6% of female total). Of the male cases of DM, males >40-65-years-old had the most cases (460, 55.2%).

**Table S6. Nutrition status in children under five.**

|  | **Female (n = 2114)** | | **Male (n = 2430)** | | **Total (N = 4544)** | |
| --- | --- | --- | --- | --- | --- | --- |
|  | **Cases** | **Prevalence, %** | **Cases** | **Prevalence, %** | **Cases** | **Prevalence, %** |
| **Well-Nourished (>13.50 cm)** | 908 | 43.0 | 1218 | 50.1 | 2126 | 46.8 |
| **At-Risk (12.60-13.50 cm)** | 782 | 37.0 | 882 | 36.3 | 1664 | 36.6 |
| **Moderate Acute Malnutrition (11.00-12.50 cm)** | 414 | 19.6 | 325 | 13.4 | 739 | 16.3 |
| **Severe Acute Malnutrition (6.30 -10.90 cm)** | 10 | 0.5 | 5 | 0.2 | 15 | 0.3 |

Nutrition status was assessed in 4,544 children under five-years-old by measurement of MUAC. 57.1% of all females were at risk for malnutrition or diagnosed with MAM or SAM compared to 49.9% of males. The prevalence of at-risk nutrition stats us 36.6% (37.0% in girls and 36.3% in boys). The prevalence of MAM was 16.3% (19.6% in girls and 13.4% in boys). The prevalence of SAM was 0.3% (0.5% in girls and 0.2% in boys). Girls were found to be more likely to be at-risk for malnutrition and also undernourished than boys.

**Table S7. BMI analysis in patients over two-years-old.**

|  | **Female (n = 25220)** | | **Male (n = 21306)** | | **Total (N = 46526)** | |
| --- | --- | --- | --- | --- | --- | --- |
|  | **Cases** | **Prevalence, %** | **Cases** | **Prevalence, %** | **Cases** | **Prevalence, %** |
| **Severely Underweight** | 2616 | 10.4 | 3471 | 16.3 | 6087 | 13.1 |
| **Underweight** | 4190 | 16.6 | 5728 | 26.9 | 9918 | 21.3 |
| **Normal weight** | 14245 | 56.5 | 10577 | 49.6 | 24822 | 53.4 |
| **Overweight** | 3383 | 13.4 | 1079 | 5.1 | 4462 | 9.6 |
| **Obese** | 786 | 3.1 | 451 | 2.1 | 1237 | 2.7 |

BMI was assessed in patients ≥ 2 years old and parameters for BMI status were derived from WHO z-scores. The mean BMI was 19.25 ± 4.75 kg/m^3^. 53.4% had normal BMI, 21.3% were underweight (between 1-2 SD from the mean), 13.1% were severely underweight (between 2-3 SD from the mean), 9.6% were overweight (between 1-2 SD from the mean), and 2.7% were obese (between 2-3 SD from the mean). Males were more severely underweight and underweight than females (16.3% vs 10.37% and 26.9% vs 16.6%, respectively). Females were more overweight and obese than males (13.4% vs 5.1% and 3.12 vs 2.1%, respectively). See Table S14 for detailed coding definitions.

**Figure S1. BMI Analysis of patients over two-years-old.**

**
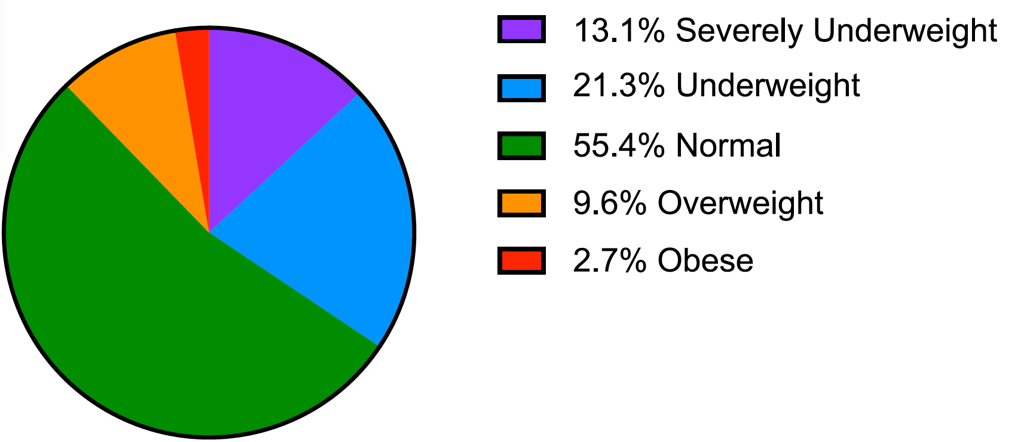
**

BMI was assessed in patients ≥ 2 years old and parameters for BMI status were derived from WHO z-scores. The mean BMI was 19.25 ± 4.75 kg/m^3^. 53.4% had normal BMI, 21.3% were underweight (between 1-2 SD from the mean), 13.1% were severely underweight (between 2-3 SD from the mean), 9.6% were overweight (between 1-2 SD from the mean), and 2.7% were obese (between 2-3 SD from the mean).

**Table S8. Anemia prevalence stratified by sex.**

|  | **Female (n = 19171)** | | | | | | **Male (n = 14381)** | | | | | |
| --- | --- | --- | --- | --- | --- | --- | --- | --- | --- | --- | --- | --- |
|  | **Anemia Grade 1** | | **Anemia Grade 2** | | **Anemia Grade 3** | | **Anemia Grade 1** | | **Anemia Grade 2** | | **Anemia Grade 3** | |
| **Age, y** | **Cases** | **Prevalence, %** | **Cases** | **Prevalence, %** | **Cases** | **Prevalence, %** | **Cases** | **Prevalence, %** | **Cases** | **Prevalence, %** | **Cases** | **Prevalence, %** |
| **10 – <18**  **(n_f_ = 2700)**  **(n_m_ = 3346)** | 84 | 3.1 | 99 | 3.7 | 7 | 0.3 | 10 | 0.3 | 1 | 0.0 | 4 | 0.1 |
| **18 – 40**  **(n_f_ = 9468)**  **(n_m_ = 5103)** | 786 | 8.3 | 665 | 7.0 | 106 | 1.1 | 36 | 0.7 | 14 | 0.3 | 12 | 0.2 |
| **>40 – 65**  **(n_f_ = 6504)**  **(n_m_ = 4790)** | 264 | 4.1 | 276 | 4.2 | 74 | 1.1 | 48 | 1.0 | 18 | 0.4 | 18 | 0.4 |
| **>65 – 80**  **(n_f_ = 445)**  **(n_m_ = 1012)** | 14 | 3.2 | 17 | 3.8 | 4 | 0.9 | 12 | 1.2 | 4 | 0.4 | 16 | 1.6 |
| **>80**  **(n_f_ = 54)**  **(n_m_ = 130)** | 2 | 3.7 | 1 | 1.9 | 0 | 0.0 | 2 | 1.5 | 0 | 0.0 | 3 | 2.3 |
| **Total** | 1150 | 6.0 | 1058 | 5.5 | 191 | 1.0 | 108 | 0.8 | 37 | 0.3 | 53 | 0.4 |

Clinical anemia was assessed in 19,171 female and 14,381 male patients by lower palpebral eyelid exam. Female patients showed a higher prevalence of anemia grades 1-3 (6.0%, 5.5%, and 1.0% compared to 0.8%, 0.3%, and 0.4%, respectively. The age group 18-40-years-old had the highest prevalence of anemia grades 1 and 2 (8.3% and 7.0%, respectively). The age group >40-65-years-old had the highest prevalence of anemia grade 3 (1.2%). In males, the age group >80-years-old had the highest prevalence of anemia grades 1 and 3 (1.5% and 2.3%, respectively. The age group >65-80-years-old was had the highest prevalence of anemia grade 2 (0.4%).

**Table S9. Presumptive TB cases, sputum samples, and confirmed diagnoses.**

| **Presumptive Cases** | | | **Number of Reports Made** | | | **Results** | |
| --- | --- | --- | --- | --- | --- | --- | --- |
| **Female** | **Male** | **Total** | **Female** | **Male** | **Total** | **Positive** | **Negative** |
| 321 | 336 | 657 | 72 | 86 | 158 | 3 | 155 |

Passive TB screening defined by cough for longer than four weeks, or two of the following symptoms: low-grade evening rise fever (LGERF), weight loss, and night sweats identified 657 presumptive cases—321 female and 336 male. Of those presumptive cases, 158 reports were made at an outside government diagnostic clinic —72 female and 86 male. There were three positive results and 155 negative results confirmed by acid-fast bacilli on sputum microscopy.

**Table S10. Areca nut consumption stratified by sex.**

|  | **Female**  **(n = 18817)** | **Male**  **(n = 13143)** | **Total**  **(N = 31960)** |
| --- | --- | --- | --- |
| **No. of Users** | 5131 | 3912 | 9043 |
| **Average Age, y** | 36.78 | 41.47 | 38.47 ± 15.50 |
| **Prevalence, %** | 27.3 | 29.8 | 28.3 |

Areca nut consumption was assessed in 31,960 patients over 15-years-old. 9043 patients (28.3%) self-reported as users with an average age of 38.47 ± 15.50 years. Male patients had a higher prevalence than female patients (29.8% and 27.3%, respectively). The average age was greater in males than females as well (41.47 years and 36.78 years, respectively.

**Table S11. Odds ratio assessing areca nut consumption and DM prevalence.**

| **+ Areca Nut, %** | **- Areca Nut, %** | **Odds Ratio** | **95% Confidence Interval** | | **p-value** |
| --- | --- | --- | --- | --- | --- |
|  |  |  | **-** | **+** |  |
| 23.07 | 16.17 | 1.55 | 1.43 | 1.70 | <0.001 |

Odds ratio analysis assessing DM prevalence with and without areca nut exposure showed that those who consumed the nut were more likely to have DM (OR: 1.55, 95%CI: 1.43-1.70, p < 0.001).

**Table S12. Regression analyses.**

|  | | **Female** | | **Male** | | **Total** | |
| --- | --- | --- | --- | --- | --- | --- | --- |
| **Dependent Variable** | **Independent Variable** | **Coefficient** | **p-value** | **Coefficient** | **p-value** | **Coefficient** | **p-value** |
| BMI | FBG | 0.12 | <0.001 | 0.13 | <0.001 | 0.11 | <0.001 |
| BMI | Systolic BP | 0.19 | <0.001 | 0.17 | <0.001 | 0.18 | <0.001 |
| DM | Systolic BP | -0.003 | <0.001 | -0.01 | <0.001 | -0.02 | <0.001 |

Regression analyses were calculated to predict BMI from FBG and systolic BP (linear regressions) and DM based on systolic BP (logistic regression). BMI showed a statistically significant positive correlation with FBG (r = 0.11, p < 0.001) and systolic BP (r = 0.18, p < 0.001). DM had a statistically significant negative correlation with systolic BP (r = -0.02, p < 0.001), however is not likely to be clinically significant.

**IV. References**

1. Lawman HG, Ogden CL, Hassink S, Mallya G, Vander Veur S, Foster GD. Comparing Methods for Identifying Biologically Implausible Values in Height, Weight, and Body Mass Index Among Youth. *Am J Epidemiol*. 2015;182(4):359-365. doi:10.1093/aje/kwv057

2. Pater C. The Blood Pressure "Uncertainty Range" - a pragmatic approach to overcome current diagnostic uncertainties (II). *Curr Control Trials Cardiovasc Med*. 2005;6(1):5. Published 2005 Apr 6. doi:10.1186/1468-6708-6-5

3. Menke A, Rust KF, Savage PJ, Cowie CC. Hemoglobin A1c, fasting plasma glucose, and 2-hour plasma glucose distributions in U.S. population subgroups: NHANES 2005-2010. *Ann Epidemiol*. 2014;24(2):83-89. doi:10.1016/j.annepidem.2013.10.008

4. Phan HTT, Borca F, Cable D, Batchelor J, Davies JH, Ennis S. Automated data cleaning of paediatric anthropometric data from longitudinal electronic health records: protocol and application to a large patient cohort. *Sci Rep*. 2020;10(1):10164. Published 2020 Jun 23. doi:10.1038/s41598-020-66925-7

5. Stoltzfus RJ, Edward-Raj A, Dreyfuss ML, et al. Clinical pallor is useful to detect severe anemia in populations where anemia is prevalent and severe. *J Nutr*. 1999;129(9):1675-1681. doi:10.1093/jn/129.9.1675
